# Ras-related C3 botulinum toxin substrate 1 activation is involved in the pathogenesis of diabetic retinopathy

**DOI:** 10.3892/etm.2014.2081

**Published:** 2014-11-19

**Authors:** YANG-JUN LI, JIE ZHANG, JING HAN, ZHAO-JIANG DU, PING WANG, YONG GUO

**Affiliations:** Department of Ophthalmology, Tangdu Hospital, The Fourth Military Medical University of the PLA, Xian, Shaanxi 710038, P.R. China

**Keywords:** Rac1, β-catenin, VE-cadherin, diabetes, retinopathy, rat, rat retinal endothelial cell

## Abstract

This study used a streptozotocin (STZ)-induced rat model of diabetes to investigate whether Ras-related C3 botulinum toxin substrate 1 (Rac1) was involved in the pathogenesis of diabetic retinopathy. The effects of Rac1 inhibition on vascular endothelial (VE)-cadherin and β-catenin expression in high glucose-induced rat retinal endothelial cells (RRECs) were additionally examined. Rac1 activation in the retinas from STZ-induced diabetic rats and in high glucose-induced RRECs was measured by reverse transcription-quantitative polymerase chain reaction analysis, immunohistochemistry and western blot analysis. The expression levels of VE-cadherin and β-catenin were also examined with or without Rac1 inhibition through small interfering (si)RNA transfection. STZ-induced diabetes was associated with an increase in the vascular permeability of the retina. Furthermore, Rac1 activation was increased in the retina of STZ-induced diabetic rats and in high glucose-induced RRECs compared with that in the controls. Immunohistochemistry showed that immunostaining of Rac1 was localized in the outer plexiform, inner nuclear, inner plexiform and ganglion cell layers and in the retinal microvasculature of rats. The expression of β-catenin was increased in the retinas of the diabetic rats at four, eight and 12 weeks after the induction of diabetes compared with that in the controls. Additionally, Rac1 activation was required for the high glucose-induced VE-cadherin expression decrease and for β-catenin expression in high glucose-induced RRECs. Rac1 inhibition by Rac1-siRNA transfection effectively prevented hyperpermeability, β-catenin expression and the VE-cadherin expression decrease in high glucose-induced RRECs. In conclusion, diabetes affects the expression of Rac1 in the retina. Rac1 may be involved in the diabetes-induced damage and/or alterations to the blood-retinal barrier through changes in VE-cadherin and β-catenin expression.

## Introduction

Diabetic retinopathy (DR) is the most common cause of visual disorders leading to irreversible blindness. Blood-retinal barrier (BRB) loss is crucial in the pathogenesis of DR; however, the precise mechanisms leading to retinal vasculature and tissue damage in DR have not been fully elucidated. The integrity of the BRB is determined by a watertight apical junctional complex that is composed of tight and adherens junctions. Adherens junctions are predominantly formed by homophilic interactions between proteins of the cadherin family, which are transmembrane calcium-dependent adhesion proteins. Vascular endothelial (VE)-cadherin is the best-characterized member of this family of proteins (1). VE-cadherin is an endothelial cell (EC)-specific adhesion molecule that connects adjacent ECs (2). While the barrier function of the endothelium is supported by multiple cell-cell adhesion systems, the disruption of VE-cadherin is sufficient to disrupt intercellular junctions (3). The association between VE-cadherin and the actin cytoskeleton is necessary for strong mechanical cell-cell interaction. This interaction is mediated via VE-cadherin-bound β- and α-catenin, which, in turn, are associated with actin filaments through actin-binding proteins, including Epithelial Protein Lost In Neoplasm, vinculin, formin-1 or α-actinin (4). The tyrosine (Tyr) phosphorylation of VE-cadherin has been reported to cause a loss of the ability of VE-cadherin to bind β-catenin (5), while the Tyr phosphorylation of β-catenin has been demonstrated to decrease the affinity of β-catenin for the cadherin and increase its turnover at junctions (5). The assembly of the VE-cadherin/catenin adhesion complex is strictly regulated; this regulation involves a number of post-transcriptional processes, including phosphorylation, dephosphorylation, protein interactions and changes in protein stability (6). Ras-related C3 botulinum toxin substrate 1 (Rac1), one of the Rho family members, has a key role in this process, which has been previously reviewed by Hall (7).

The active, guanosine triphosphate (GTP)-bound form of Rac1 regulates the formation of submembraneous actin cytoskeleton structures, which leads to the formation of lamellipodia (7). Rac1 localization at membrane sites is a critical event in the maturation of epithelial cell adherens junctions during cell polarization (8). It has previously been demonstrated that the Rac1-induced generation of reactive oxygen species (ROS) disrupts VE-cadherin-based cell-cell adhesion (9). Furthermore, the interaction of active Rac1 with IQ motif containing GTPase activating protein 1 (IQGAP1), which is associated with a disassembly of E-cadherin-mediated adherens junctions, has been shown to destabilize E-cadherin-mediated cell-cell adhesion in pancreatic carcinoma cells. By contrast, the inhibition of Rac1 activity increased E-cadherin-mediated cellular adhesion (10). Rac and ROS are required for the regulation of vascular endothelial growth factor (VEGF)-induced microvascular permeability (11). Furthermore, the nuclear accumulation of β-catenin in response to Wnt requires Rac1 activation. A previous study demonstrated that silencing of the Rac1 gene in the mouse embryonic limb bud ectoderm disrupted canonical Wnt signaling and phenocopy deletion of β-catenin, causing severe truncations of the limb (12). However, to date, no biological function of Rac1 and β-catenin in DR has been demonstrated.

## Materials and methods

### Animal models

A total of 70 male Sprague Dawley rats with body weights of 260±5.6 g were obtained from the Laboratory Animal Center of the Fourth Military Medical University of the PLA (Xian, China) for use in this study. Of these, 40 animals were housed under a 12-h light/dark cycle, with four rats per cage, and were fed standard rat chow and water *ad libitum*. Diabetes was induced by a single intraperitoneal injection of streptozotocin (STZ; Sigma, St. Louis, MO, USA) in 0.05 M citrate buffer (pH 4.5) at a dose of 65 mg/kg body weight, and was defined as blood glucose levels >15 mmol/l (270 mg/dl), seven days after STZ injection. The other 30 animals received a single intraperitoneal injection of 0.05 M citrate buffer as a control group. Blood samples were obtained from the rat tail vein and the glucose concentration was determined with an automatic analyzer (Glucometer Elite XL; Bayer Inc., Toronto, ON, Canada) using glucose oxidase/potassium ferricyanide reagent strips. Body weights and blood glucose levels were noted once a week after the induction of diabetes. This study was carried out in strict accordance with the recommendations in the Guide for the Care and Use of Laboratory Animals of the National Institutes of Health. The animal use protocol was reviewed and approved by the Institutional Animal Care and Use Committee of the Fourth Military Medical University of the PLA.

### Measurement of retinal vascular permeability

Retinal vascular permeability was quantified by measuring albumin leakage from blood vessels into the retina using the Evans blue method in accordance with the procedures described in a previous study (13) with minor modifications (14).

### Tissue and retinal digest preparation for immunohistochemistry

The animals were sacrificed with an intraperitoneal overdose of pentobarbital. The eyes were enucleated, fixed in 4% paraformaldehyde for 24 h and rinsed with water for 24 h. The retinas and tissues were carefully removed. For retinal immunohistochemistry, the fixed retinas were embedded in paraffin and 5-μm histological sections were made.

For retinal digest preparation, the retinal vasculatures of rats were isolated as described previously (15). Briefly, each fixed retina was cut into four parts and rinsed with 0.1 M phosphate-buffered saline (PBS) for 24 h, prior to being placed into 3% pancreatin solution dissolved by 0.1 mol/l Tris-HCl buffer fluid (pH 7.8) and incubated for 3 h at 37°C. The solution was changed only when the retina was not completely digested. The samples were then moved to distilled water and agitated gently until the inner membrane and remaining neurosensory retina were completely rinsed off and only a thin layer of retinal vascular network was left. The retinal vascular network layer was washed out and carried onto a slide, prior to being unfolded and dried naturally. The samples were stored at −20°C in preparation for immunohistochemistry.

### Cell culture

RRECs were purified as previously described (16). Contamination of the microvessel preparations by neuronal tissue, assessed subsequent to microscopic examination and western blotting with a monoclonal antibody raised against rhodopsin (Santa Cruz Biotechnology, Inc., Santa Cruz, CA, USA), was typically <5% (6). The RRECs were isolated and cultured as previously described (6). The RRECs were grown in primary culture on dishes coated with collagen IV/fibronectin (2 and 4 μg/cm^2^, respectively; Sigma-Aldrich Corp., St. Louis, MO, USA) in Dulbecco’s modified Eagle’s medium (DMEM; Vector Laboratories, Inc., Burlingame, CA, USA) containing 15% human serum, 80 μg/ml heparin, 2 mm glutamine and antibiotics (penicillin G potassium and streptomycin sulphate). The purity of the RREC cultures was assessed, in accordance with a previously described method (6), to be >90%. The media were supplemented with 10 μM unesterified docosahexaenoic acid (DHA) at a molar ratio over albumin in serum of 1:10. This supplementation restored the DHA proportion of these cells to the original value observed in the intact microvessels (6). At confluence, the RRECs were trypsinized and seeded in gelatin- and fibronectin-coated dishes (Sigma-Aldrich Corp.). An identical batch of cells derived from one primary culture was used to compare the effects of hyperglycemic conditions. Glucose or mannitol (15, 25 or 30 mm) or bovine serum albumin (control) was added to the RREC culture medium.

### Recombinant pSUPER-Rac1-shRNA construction

The expression vector, pSUPER-GFP/Neo RNA interference (RNAi) system (Oligoengine, Seattle, WA, USA) was used for the expression of siRNA. The selection of the human and rat homologous Rac1 gene siRNAs was based on the characterization of siRNA by Elbashir *et al* (17). Among the different Rac1 target sequences examined, the 19-nucleotide gene-specific sequence spanning between nucleotides 367 and 385 downstream of the gene transcription start site was selected to suppress Rac1 gene expression. Following Basic Local Alignment Search Tool analysis (www.ncbi.nlm.nih.gov/BLAST/), to ensure that there was no significant sequence homology with other human and rat genes, the selected sequence was AGACACGATCGAGAAACTG. The short hairpin (sh)RNA insert template oligonucleotides consisted of an RNA duplex containing a sense strand (5′-GAT CCCCAGACACGATCGAGAAACTGTTCAAGAGACAGT TTCTCGATCGTGTCTTTTTTGGAAA-3′) and an antisense strand (5′-AGCTTTTCCAAAAAAGACACGATCGAGAAA CTGTCTCTTGAACAGTTTCTCGATCGTGTCTGGG-3′). The template oligonucleotides were synthesized and purified by Sangon Biotech Company (Shanghai, China), and then annealed and ligated into the *Bgl*II and *Hin*dIII sites of the linearized pSUPER-GFP/Neo RNAi system. A non-silencing control vector (non-silencing shRNA; NS) was constructed using a 19-nucleotide sequence (GCGCGCTTTGTAGGATTCG) with no significant homology to any mammalian gene sequence. All inserted sequences were confirmed by DNA sequencing.

### siRNA transfection

RRECs were cultured at 37°C in a 5% CO_2_ atmosphere in DMEM supplemented with 10% fetal calf serum. Cells were plated in 24-well plates at 2×10^5^ cells per well, cultured for 24 h and then transfected with pSUPER-Rac1-shRNA according to the manufacturer’s instructions (Oligoengine). Negative control cells were treated with NS consisting of a circle plasmid encoding an siRNA whose sequence was not found in mouse, human or rat genome databases. Lipofectamine 2000™ (Invitrogen Life Technologies, Carlsbad, CA, USA) was used for the transfection according to manufacturer’s instructions. Seventy-two hours after transient transfection, silencing was examined using western blotting and reverse transcription-quantitative polymerase chain reaction (RT-qPCR) analysis.

### [^3^H]Sucrose permeability assay

The [^3^H]sucrose permeability assay was performed as described by Kim *et al* (18), with minor modifications. In brief, RRECs (1×10^5^ cells) with or without Rac1 gene siRNAs, NS siRNA or siRNA targeting Rac1 were plated onto a Transwell™ filter. At 60–70% confluency the complete medium was replaced with endothelial basal medium-2 (EBM™-2; Lonza, Basel, Switzerland) containing 25 mm D-glucose for 24 h. [^3^H]Sucrose, 50 μl (0.8 μCi/ml) (1 μCi/μl; Amersham Pharmacia Biotech, Amersham, UK), was added to the upper compartment. The amount of radioactivity that diffused into the lower compartment was determined after 30 min by a liquid scintillation counter (Perkin Elmer/Wallac, Inc., Gaithersburg, MD, USA).

### Total RNA extraction and qPCR

Total RNA was isolated from the RRECs and retinas at passage 17 using Trizol™ reagent (Gibco-BRL, Gaithersburg, MD, USA) according to the manufacturer’s instructions. A total of 2 μg isolated RNA was reverse-transcribed into cDNA using a high-capacity cDNA synthesis kit (Takara Bio, Inc., Shiga, Japan). Quantitative analysis of gene expression was generated using a sequence detection system (7300 Real-Time; Applied Biosystems, Foster City, CA, USA) and a SYBR Green Real-Time PCR Master Mix kit (Takara Bio, Inc). Semi-log amplification curves were evaluated by the comparative quantification method (2^−ΔΔCt^), and β-actin was used for data normalization. The primer sequences (synthesized by Sangon Biotech Company) used in the present study were as follows: Rac1 forward, 5′-GGACAAGAAGATTAT GACAG-3′ and reverse, 5′-ATACCACTTTGCACGGACAT-3′; VE-cadherin forward, 5′-CCTACCAGCCCAAAGTGTGT-3′ and reverse, 5′-GACTTGGCATCCCATTGTCT-3′; β-catenin forward, 5′-TGGGCAGTTTGCAATGACCAGA-3′ and reverse, 5′-ACGCATAATAGCATGGCGGGAA-3′; β-actin forward, 5′-GAGGGAAATCGTGCGTGAC-3′ and reverse, 5′-GAGTGACAGGTGGAAGGTC-3′.

### Immunohistochemistry

The paraffin-embedded retinal tissue sections were rehydrated through xylene and graded alcohols. These rehydrated retinal tissue sections or slides containing the retinal vasculature were treated with 3% hydrogen peroxide for 10 min. The retinal tissue sections were made by microwaving in sodium citrate for 20 min for antigen retrieval. Subsequent to three 5-min rinses in PBS and incubation with normal blocking serum (Vector Laboratories, Inc.) for 30 min, the retinal tissue sections and slides containing the retinal vasculature were incubated with primary antibodies in block solution overnight. The primary antibodies were rabbit anti-GTP-Rac1 (diluted 1:50; Stressgen Biotechnologies Corp., Victoria, BC, Canada), rabbit anti-VE-cadherin (1:100; Santa Cruz Biotechnology, Inc.) and rabbit anti-β-catenin polyclonal antibody (diluted 1:200; Abcam, Cambridge, UK). Subsequent to a further three 5-min rinses in PBS, the samples were incubated with a 1:2,000 dilution of biotinylated goat anti-rabbit immunoglobulin G (IgG) antibody (Vector Laboratories, Inc.) for 30 min and for 30 min with horseradish peroxidase-conjugated avidin (Vector Laboratories, Inc.). The antigens were detected with a diaminobenzidine kit (Sigma, St. Louis, MO, USA); the brown/yellow reaction product was visualized by light microscopy. Negative controls consisted of incubations in 5% isotype control serum without the primary antibody and did not generate a reaction product. For image capture, a high-resolution video camera (DXC-960MD; Sony Corp., Tokyo, Japan) mounted on a BH-2 Olympus microscope (Olympus Corp., Melville, NY, USA) was computer-linked and retinal images were selected at a distance of ~0.8 mm from the optic nerve head.

### Western blot analysis

Western blotting was performed using standard western blotting methods. The protein concentration was measured using a bicinchoninic acid protein assay kit (Pierce, Rockford, IL, USA). Equal amounts of protein were separated by 5–10% sodium dodecyl sulfate-polyacrylamide gel electrophoresis and transferred electrophoretically onto nitrocellulose membranes (Amersham Pharmacia Biotech). The membranes were blocked for 30 min in 5% skimmed milk. Following blocking, the membranes were incubated overnight with anti-VE-cadherin (1:1,000; Santa Cruz Biotechnology, Inc.), anti-GTP-Rac1 (1:1,000; Zymed Laboratories, San Francisco, CA, USA) and anti-β-catenin (1:2,000; Zymed Laboratories) antibodies at 4°C. Following washing with PBS with Tween 20 (PBS-T), the membranes were incubated for 1 h at room temperature with horseradish peroxidase-conjugated anti-rabbit IgG or anti-mouse IgG (1:10,000, Pierce) in PBS-T and 1% skimmed milk. To ensure the equal loading of protein in each lane, the blots were stripped and reprobed with an antibody against β-actin. Intensity values were normalized relative to control values. The blots were scanned using a flatbed scanner and the band intensity was analyzed using the TINA software program (Raytest, Staubenhardt, Germany).

### Rac1 activity assays

RRECs were cultured at 37°C in EBM-2 supplemented with EGM™-2 SingleQuots™ (Lonza). RRECs were used between passages four and eight. At 60–70% confluency the medium was changed to EBM-2 (without supplements) containing 25 mM D-glucose for the indicated time periods. Cells incubated with 5 mM D-glucose plus 20 mM D-mannitol or 25 mM L-glucose served as controls. Following stimulation the cells were kept on ice, washed with ice-cold PBS and assayed for Rac1 activation with glutathione S-transferase-p21-activated kinase 1B (GST-PAK1B), as described by Sander *et al* (19). The beads were washed four times with lysis buffer and, after the final wash, resuspended in sample buffer. Samples were then analyzed by western blotting.

### Statistical analysis

Data are presented as the mean ± standard deviation. Comparisons between two groups were conducted with an independent-samples t-test. One-way analysis of variance was used for multiple comparisons. P<0.05 was considered to indicate a statistically significant difference.

## Results

### Body weight change and blood glucose level

**Figure 1:**
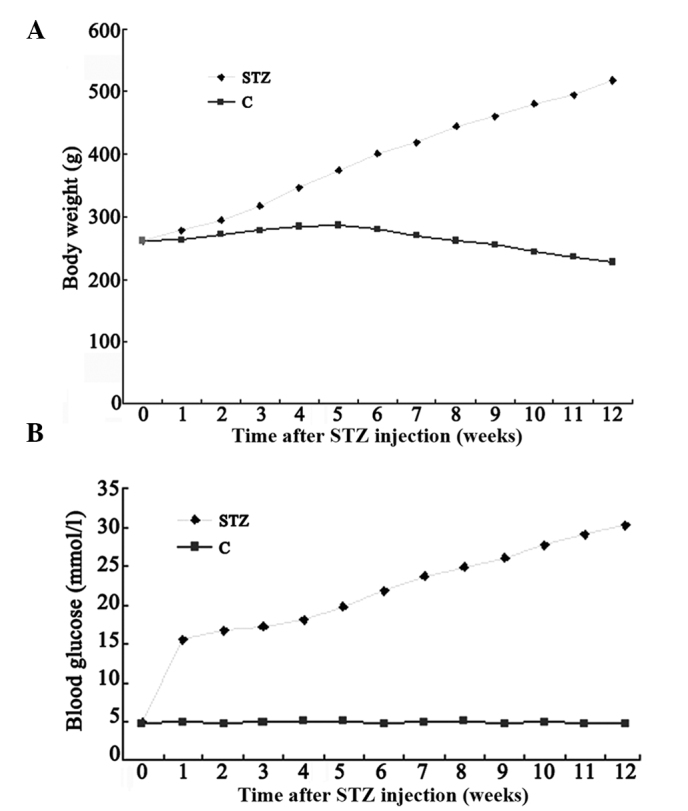
Variations in (A) body weights and (B) blood glucose levels in the control and STZ-induced diabetic rats. STZ, streptozotocin; C, control.

In the STZ-induced diabetic rats, body weight was significantly lower than that in the age-matched controls. At 12 weeks after induction of diabetes, the body weight of the diabetic rats was 236±14.1 g (n=40), which was significantly decreased (P<0.001) from the body weight of the controls (496±10.2 g, n=30) (Fig. 1A).

The diabetic rats showed significant increases in blood glucose levels compared to the control rats. At 12 weeks after induction of diabetes, the blood glucose level of the diabetic rats was 30.2±4.8 mmol/l (n=40), which was significantly different (P<0.001) from that of the controls (4.9±0.12 mmol/l, n=30) (Fig. 1B).

### Alteration of retinal vascular permeability in STZ-induced diabetic rats

**Figure 2:**
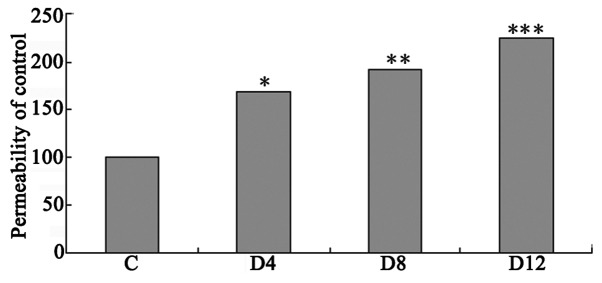
Time course of the diabetes-induced increase in vascular permeability. Vascular permeability was measured at four, eight and 12 weeks after the induction of diabetes (D4, D8 and D12, respectively). Vascular permeability was normalized by the total protein concentration in the retina and expressed as a percentage of the control (n=5). ^*^P<0.005 vs. the control; ^**^P<0.005 vs. the control and D4; ^***^P<0.005 vs. the control, D4 and D8.

Vascular permeability in the retina was measured using the Evans blue method. Vascular permeability was increased by 68, 91 and 125%, respectively (each P<0.005), in the retinas at four, eight and 12 weeks after the induction of diabetes compared with that in the controls (Fig. 2).

### Activity and expression of Rac1 in the retina of STZ-induced diabetic rats

**Figure 3:**
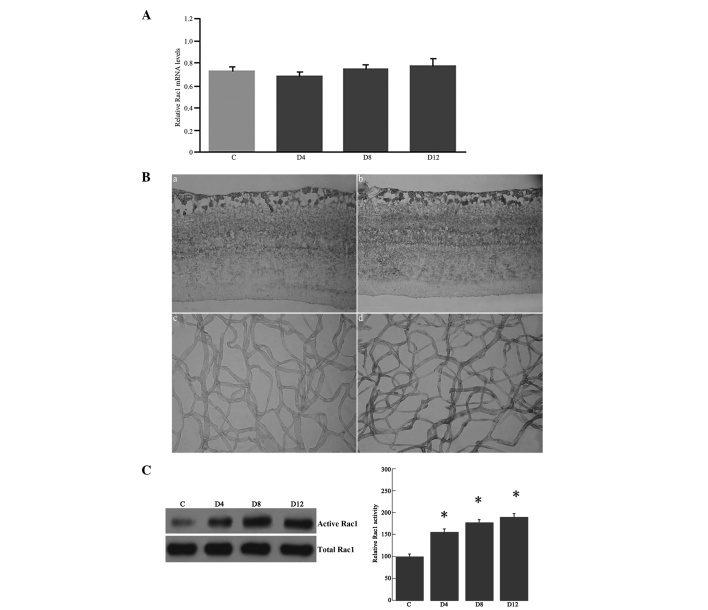
(A) Reverse transcription-quantitative polymerase chain reaction analysis of Rac1 mRNA expression in the retinas of streptozotocin-induced diabetic and control rats. The retinal Rac1 mRNA expression was examined in the rat retinas at four, eight and 12 weeks after induction of diabetes (D4, D8 and D12, respectively), as well as in the controls, using β-actin as an internal control. Levels of Rac1 mRNA expression remained unchanged in the diabetic retinas at four, eight and 12 weeks after induction of diabetes compared with those in the controls. (B) Immunohistochemical analysis of Rac1 in the retinas and retinal vasculature isolated by the trypsin digest technique: (a) Control and (b) diabetic rat retinas; (c) control and (d) diabetic rat retinal vasculature. The immunostaining of Rac1 was present in the outer plexiform, inner plexiform and ganglion cell layers and in the endothelial cells and pericytes. Rac1 immunoreactivity was increased in the retinas and retinal vasculature of diabetic rats at 12 weeks after the induction of diabetes (b and d) compared with that of the controls (a and c). In the negative control staining, no immunoreactivity for Rac1 was found in the retinas and the retinal vasculature (figure not shown) (original magnification, ×400). (C) Rac1 activity from the retinal lysates of the rats. Autoradiography depicting the Rac1 activity shows increased Rac1 activity in the retinas at four, eight and 12 weeks after the induction of diabetes compared with that in the controls. ^*^P<0.05 vs. the control. C, control; Rac1, Ras-related C3 botulinum toxin substrate 1.

To examine whether chronically elevated blood glucose levels affected the expression of Rac1, RT-qPCR experiments were performed using β-actin for normalization. The results showed that the level of Rac1 mRNA expression was not increased in the diabetic retinas at four, eight and 12 weeks after induction of diabetes compared with that in the controls (Fig. 3A).

Immunohistochemical analysis using anti-Rac1 monoclonal antibody showed that the immunostaining of Rac1 occurred in the retinal vasculature, including the ECs and pericytes, and in the outer plexiform layer (OPL), the inner nuclear layer (INL), the inner plexiform layer (IPL) and the ganglion cell layer (GCL) of the rat retinas; the Rac1 immunoreactivity was significantly increased in the retinas at 12 weeks after the induction of diabetes compared with that in the controls (Fig. 3B).

The Rac1 activity in the retinas at various time-points was then determined in the STZ-induced diabetic and control rats using the CRIB-domain of PAK1B (GST-PAK) as an activation-specific probe for activated Rac1, as described previously (20). As shown in Fig. 3C, Rac1 activation increased by 55.6, 77.6 and 89.8%, respectively (each P<0.05), in the retinas at four, eight and 12 weeks after the induction of diabetes compared with that in the controls.

### Level of β-catenin expression

**Figure 4:**
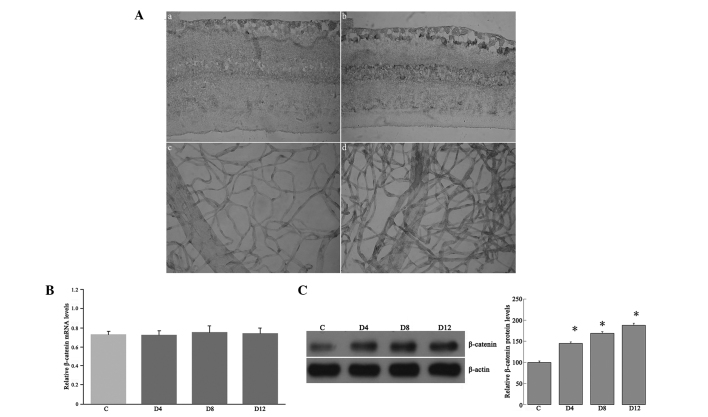
(A) Representative immunohistochemical analysis of β-catenin in the retinas and retinal vasculature. β-catenin immunoreactivity was observed in the retinal vasculature and in the outer plexiform, inner nuclear, inner plexiform and ganglion cell layers of retinas from (a and c) control rats and (b and d) diabetic rats 12 weeks after the induction of diabetes; immunoreactivity was increased in the retinas of the diabetic rats. In the negative control staining, no immunoreactivity for β-catenin was found in the retinas and retinal vasculature (figure not shown) (original magnification, ×400). (B) Reverse transcription-quantitative polymerase chain reaction analysis of β-catenin mRNA expression in the retinas of streptozotocin-induced diabetic and control rats. The retinal β-catenin mRNA expression was examined in the rat retinas at four, eight and 12 weeks after the induction of diabetes (D4, D8 and D12, respectively), as well as in the controls, using β-actin as an internal control. (C) Western blot analysis for β-catenin from the retinal lysates of the rats. Autoradiography depicting the β-catenin shows the β-catenin expression increased in the retinas at four, eight and 12 weeks after the induction of diabetes compared with that in the controls. ^*^P<0.05 vs. the control.

To further evaluate the effects of hyperglycemia on β-catenin in the retinas from the STZ-induced diabetic rats, its immunoreactivity was determined using anti-β-catenin monoclonal antibody. The results indicated that immunostaining of β-catenin was present in the retinal vasculature and in the OPL, INL, IPL and GCL of the retina in both the diabetic rats at 12 weeks after the induction of diabetes and the controls; however, the β-catenin immunoreactivity was significantly increased in the retinas at 12 weeks after the induction of diabetes compared with that in the controls (Fig. 4A).

To examine the effects of hyperglycemia on the signaling molecules of β-catenin, the activity and expression of β-catenin was determined by RT-qPCR and western blot analysis. The RT-qPCR showed that levels of β-catenin mRNA expression were not changed in the retinas of the diabetic rats at four, eight or 12 weeks after the induction of diabetes compared with that in the controls (Fig. 4B); however, western blotting showed that β-catenin protein levels were increased by 45.2, 68.8 and 88.1%, respectively (each P<0.05), in the retinas at four, eight and 12 weeks after the induction of diabetes compared with that in the controls (Fig. 4C).

### High glucose-induced increased Rac1 activity in RRECs

**Figure 5:**
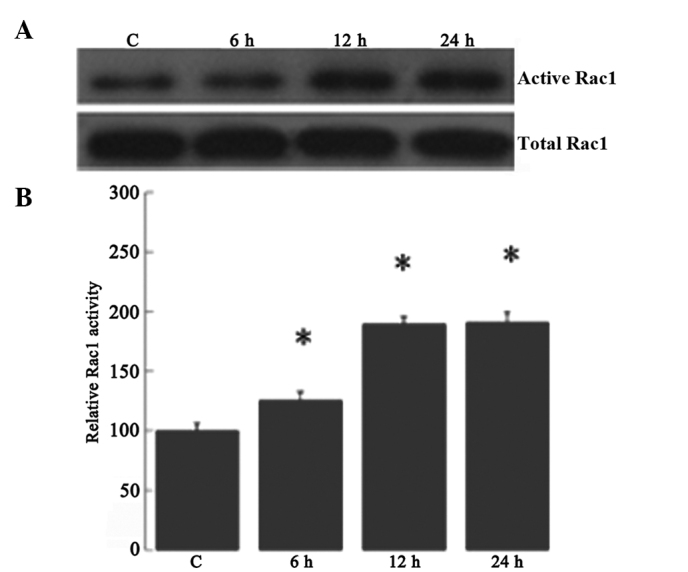
Rac1 activity in high glucose-induced RRECs. At 70% confluency, the complete medium was replaced with endothelial basal medium-2 (without supplements) for 2 h, and the RRECs were subjected to high glucose levels for 12 h and analyzed for Rac1 activity by (A) western blot analysis. β-actin served as the loading control. (B) Autoradiography depicting the Rac1 activity shows increased Rac1 activity in the high glucose-induced RRECs at six, 12 and 24 h compared with that in the controls. Each point represents the mean ± standard deviation of three independent experiments, each performed in triplicate. ^*^P<0.05 vs. the control. C, control; Rac1, Ras-related C3 botulinum toxin substrate 1; RREC, rat retinal endothelial cell.

It was next investigated whether high glucose regulates Rac1 activity in RRECs. The Rac1 activity in high glucose-induced RRECs at various time-points was determined using the CRIB-domain of PAK1B (GST-PAK) as an activation-specific probe for activated Rac1, as described previously (19). Rac1 activity was increased by 25.6, 89.8 and 90.8%, respectively, in the high glucose-induced RRECs at six, 12 and 24 h compared with that in the controls (Fig. 5).

### Effect of Rac1 activity on the permeability of high glucose-induced RRECs

**Figure 6:**
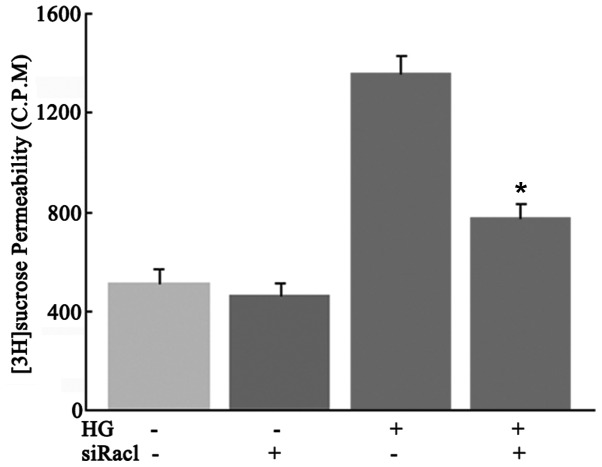
Inhibition of Rac1 by Rac1-siRNA attenuates high glucose-induced hyperpermeability in RRECs. RRECs with or without transfection of Rac1-siRNA were treated with 25 mm glucose for 12 h. The [^3^H]sucrose permeability assay in RRECs was measured as counts per minute (C.P.M.). Each point represents the mean ± standard deviation of three independent experiments, each performed in triplicate (n=6). ^*^P<0.05 vs. high glucose-induced RRECs without Rac1-siRNA transfection. Rac1, Ras-related C3 botulinum toxin substrate 1; RREC, rat retinal endothelial cell; siRNA, small interfering RNA; HG, high glucose.

To investigate the effect of Rac1 inhibition by Rac1-siRNA on high glucose-induced hyperpermeability in RRECs, a [^3^H]sucrose permeability assay was performed. As shown in Fig. 6, Rac1 inhibition by Rac1-siRNA transfection effectively prevented hyperpermeability in high glucose-induced RRECs (P<0.05).

### Effect of Rac1 activity on VE-cadherin and β-catenin protein expression

**Figure 7:**
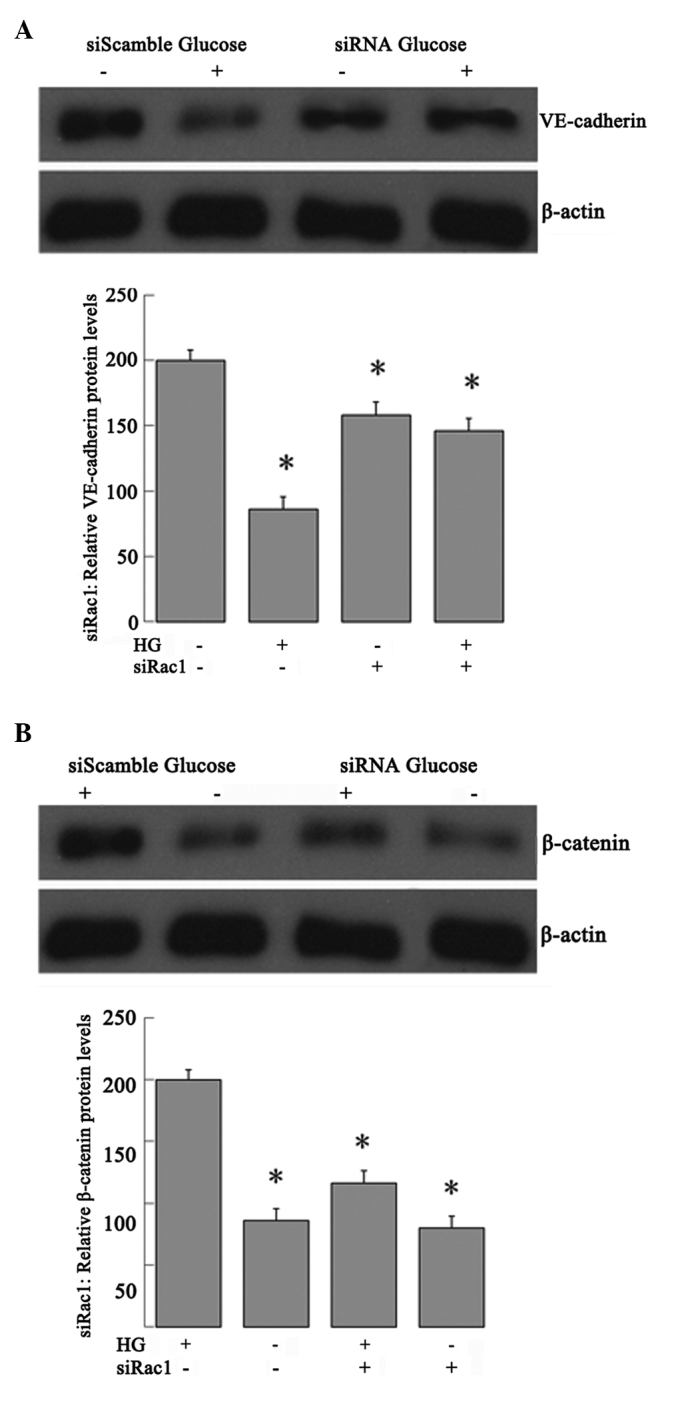
(A) Effects of Rac1 activity on VE-cadherin expression. At 70% confluency, the complete medium was replaced with EBM™-2 (without supplements) for 2 h. RRECs with or without transfection of Rac1-siRNA for 48 h were treated with high glucose for 12 h and analyzed for VE-cadherin expression by western blot analysis (n=6). β-actin served as the loading control. Quantitative analysis was performed by measuring protein expression relative to the control. Each point represents the mean ± standard deviation of three independent experiments, each performed in triplicate. High glucose-induced VE-cadherin expression was shown to be Rac1-dependent. ^*^P<0.05 vs. the control (HG^−^ and Rac1-siRNA^−^). (B) Effect of Rac1 activity on β-catenin expression. At 70% confluency, the complete medium was replaced with EBM-2 (without supplements) for 2 h. RRECs with or without transfection of Rac1-siRNA (multiplicity of infection 0.5 for 48 h) were treated with high glucose for 12 h and analyzed for β-catenin expression by western blot analysis (n=6). β-actin served as the loading control. Quantitative analysis was performed by measuring protein expression relative to the control. Each point represents the mean ± standard deviation of three independent experiments, each performed in triplicate. High glucose-induced β-catenin expression was shown to be Rac1-dependent. ^*^P<0.05 vs. the control (HG^−^ and Rac1-siRNA^−^). EBM-2, endothelial basal medium-2; Rac1, Ras-related C3 botulinum toxin substrate 1; RREC, rat retinal endothelial cell; siRNA, small interfering RNA; HG, high glucose.

To further evaluate the effects of Rac1 activity on VE-cadherin and β-catenin protein expression in the high glucose-induced RRECs, the RRECs were transfected with Rac1-siRNA or NS siRNA for 12 h and then treated with 25 mmol/ml glucose. Western blot analysis showed that Rac1 was required for the high glucose-induced VE-cadherin expression decrease (Fig. 7A) and also for high glucose-induced β-catenin expression (Fig. 7B). Cells transfected with the NS siRNA were able to decrease VE-cadherin expression and enhance β-catenin expression upon high-glucose treatment.

## Discussion

Retinopathy is one of the most disabling diabetic complications, characterized by functional abnormalities of the retinal microvasculature. In the eyes, the BRB has an important role in retinal homeostasis; breakdown of the BRB in pathological conditions such as diabetes may lead to retinopathy and blindness. In this process, hyperglycemia is an underlying contributing factor; however, the mechanisms that mediate the BRB breakdown are not yet fully understood in DR. The present results demonstrated for the first time, to the best of our knowledge, that Rac1 activity and β-catenin expression were increased in the early stages of DR. Furthermore, Rac1 inhibition decreased β-catenin expression and prevented the decrease in VE-cadherin expression in high glucose-induced RRECs. These findings may enhance the understanding of the pathogenesis of DR.

The BRB breakdown eight days after the induction of diabetes by STZ occurs predominantly at the level of the retinal blood vessels (20). In agreement with this, the present results showed that retinal permeability increased gradually in the diabetic retinas with the progression of DR. Similarly, western blot analysis showed that Rac1 activity increased gradually in the diabetic retinas with the progression of DR. This suggests that increased Rac1 activity may be involved in the retinal control and development of vascular permeability in STZ-induced DR. The present results demonstrated that increased Rac1 activity occurred in the retina at four, eight and 12 weeks after the induction of diabetes by STZ, although Rac1 mRNA levels remained unchanged. The results also revealed that the immunoreactivity of Rac1 was increased in the retinas of the STZ-induced diabetic rats. Immunostaining of Rac1 was localized to the cells in the OPL, the INL, the IPL, the GCL and the microvessels of the rat retinas. It has been suggested that Rac1 reorganizes the VEGF-induced actin cytoskeleton in ECs by regulating nicotinamide adenine dinucleotide phosphate-oxidase-derived ROS during EC migration (21). The activation of Rac1 in choroidal ECs is essential for their migration across a monolayer of retinal pigment epithelium towards a VEGF gradient (22). These results suggest that Rac1 and VEGF interact reciprocally through an ROS-dependent signaling pathway.

Appropriate levels of active Rac are required for the formation and maintenance of adherens junctions, and either too high or too low activities promote disassembly. Both constitutively active and dominant negative Rac increase endothelial permeability, consistent with the necessity of maintaining levels of Rac within strict limits for optimal junctional integrity (23). In addition, several studies have demonstrated that the activation of Rac1 downstream of VEGF, and other growth factors, promotes junction disassembly and increases permeability (24,25). VEGF has been observed to regulate EC permeability through PAK, a direct downstream effector of Rac (26); however, this association is more complex than simply high Rac activation leading to increased permeability. That active Rac can both increase and decrease permeability depending on the stimulus leads to the hypothesis that the route of activation may be critical and that different scaffolding proteins may direct Rac signaling pathways in different directions. A previous study showed that Rac1 gene-targeting shRNA successfully inhibited hypoxia-induced retinal neovascularization and VEGF expression in a mouse model of oxygen-induced retinopathy (27). In a different study, it was demonstrated that VEGF regulated microvascular permeability through the activation of Rac1 and the production of ROS. These molecules, in turn, regulated the Tyr phosphorylation of adherens junction proteins VE-cadherin and β-catenin, ultimately regulating junctional integrity (11). The present results showed that Rac1 inhibition by Rac1-siRNA transfection effectively prevented hyperpermeability, the decrease in VE-cadherin expression and the increase in β-catenin protein levels in high glucose-induced RRECs.

The association of VE-cadherin with the actin cytoskeleton is necessary for strong mechanical cell-cell interaction, which is mediated via E-cadherin-bound β-catenin. The assembly of the VE-cadherin/catenin adhesion complex is under tight control, which involves different post-transcriptional processes, including phosphorylation and dephosphorylation, protein interactions and the alteration of protein stability (6). Published data have shown that the VE-cadherin expression decreases in rats after two weeks of diabetes (28). The enhanced phosphorylation of VE-cadherin at Tyr-731 changes the binding affinity for β-catenin and likely decreases cytoskeletal attachment (5). The loss of binding affinity for VE-cadherin and β-catenin, which may change the phosphorylation of β-catenin, may affect the levels of β-catenin protein. The present results indicated that the immunostaining of β-catenin was present in the retinal vasculature and in the OPL, INL, IPL and GCL of the retina in both the diabetic rats at 12 weeks after the induction of diabetes and the controls, but the β-catenin immunoreactivity was significantly increased in the retinas of the rats at 12 weeks after the induction of diabetes. Although the data showed that levels of β-catenin mRNA were not increased in the diabetic retinas at four, eight and 12 weeks after the induction of diabetes, western blot analysis showed that β-catenin protein levels were increased in the retinas following the induction of diabetes. In a previous study, it was shown that retinal levels and nuclear translocation of β-catenin were increased in humans with DR and in three DR models. The high glucose-induced activation of β-catenin was attenuated by aminoguanidine, suggesting that oxidative stress is a direct cause for the Wnt pathway activation in diabetes (29). Following its translocation into the nucleus, β-catenin acts as a transcription factor. Under normal physiological conditions in ECs, cytosolic β-catenin binds to a protein complex, such as glycogen synthase kinase 3β (GSK3β) (31). GSK3β-dependent phosphorylation of β-catenin leads to β-catenin ubiquitination and degradation. When GSK3β is phosphorylated it loses its activity; thus, β-catenin escapes ubiquitination, accumulates in the cytosol and translocates into the nucleus to induce gene transcription.

The important role of the Rho family member Rac1 in the VE-cadherin/catenin adhesion complex, which is tightly controlled and requires the regulated assembly of actin filaments at sites of cell-cell contacts, has been reviewed by Hall (7). Data show that the enhanced expression of active Rac1 reduces cell-cell adhesion and increases directed cell motility and migration through the extracellular matrix. Furthermore, activated Rac1 binds to IQGAP1, which is linked to a reduced association of IQGAP1 with β-catenin. These alterations have been shown to be associated with a disassembly of the E-cadherin/catenin adhesion complex, resulting in inhibited cellular aggregation and an elevated migratory capacity of pancreatic carcinoma cells (10). The present results showed that Rac1 activation inhibited VE-cadherin protein expression and increased the expression of β-catenin protein levels in high glucose-induced RRECs. In addition, Rac1 inhibition by Rac1-siRNA transfection effectively prevented the decrease in VE-cadherin expression and increase in β-catenin protein levels in high glucose-induced RRECs. The present data provide novel insight that Rac1 activation is involved in BRB breakdown in diabetes.

In conclusion, in the present study a pathway by which Rac1 activation increased endothelial permeability was identified, demonstrating that Rac1 inhibits VE-cadherin protein expression and increases β-catenin protein expression. Rac1 activation has been implicated in numerous pathological situations where vascular permeability is altered.
